# P-1030. Clinical Profile and Outcomes of Patients with Central Line-Associated Bloodstream Infection among Admitted Patients in a Tertiary Hospital in the Philippines

**DOI:** 10.1093/ofid/ofaf695.1226

**Published:** 2026-01-11

**Authors:** Charisma Marie S Sy, Kingbherly L Li

**Affiliations:** Chinese General Hospital and Medical Center, Quezon City, National Capital Region, Philippines; University of the Philippines Manila- Philippine General Hospital, Metro Manila, National Capital Region, Philippines

## Abstract

**Background:**

Central line-associated bloodstream infection (CLABSI) is a preventable laboratory-confirmed bloodstream infection in a patient with a central venous catheter without another attributable source of infection associated with increased hospital costs and mortality. In the Philippines, there is not much published literature documenting CLABSI in the local hospital setting. This study primarily aimed to describe the clinical presentation, profile, and outcome of patients diagnosed with CLABSI.

**Figure:**
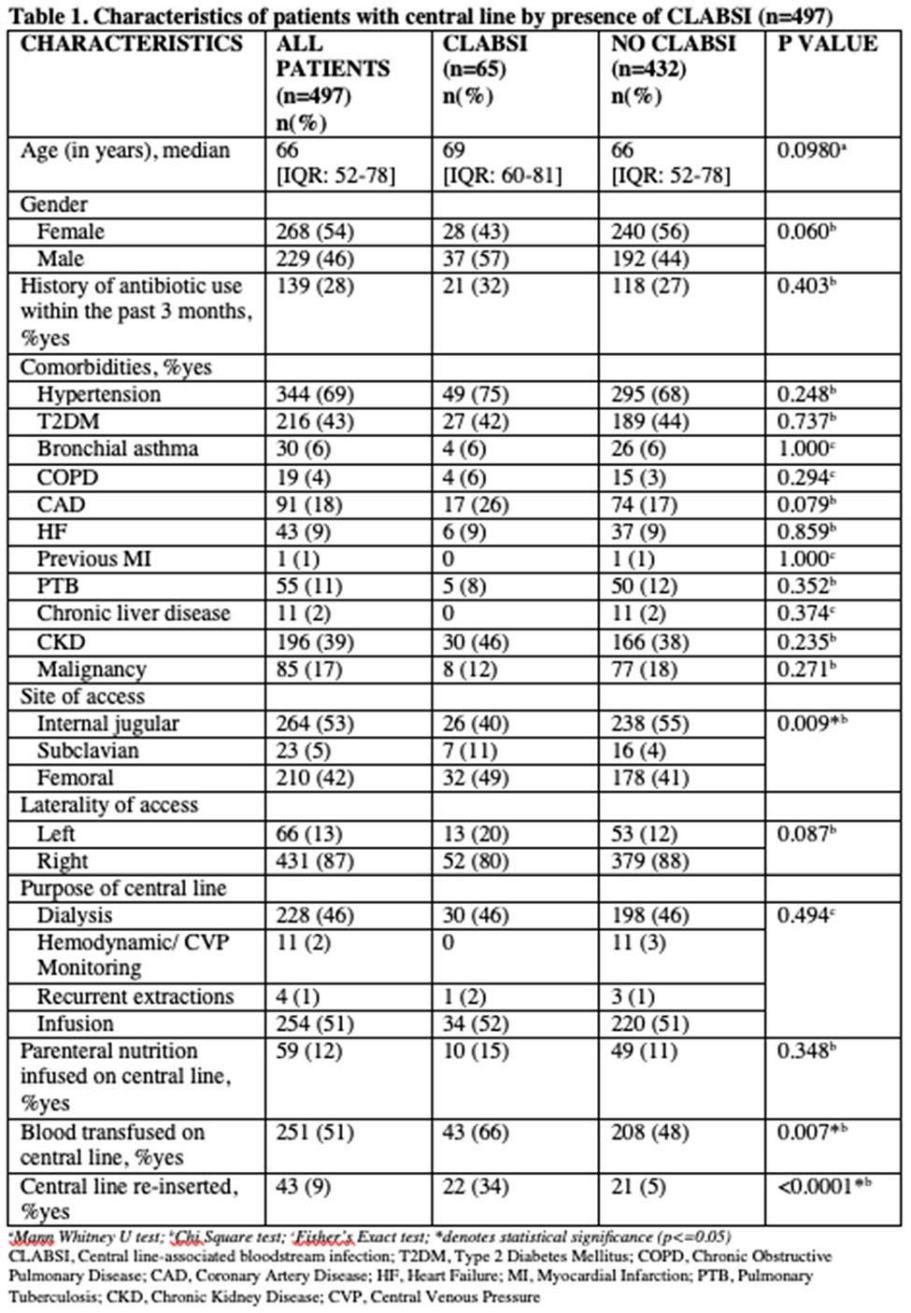

**Figure:**
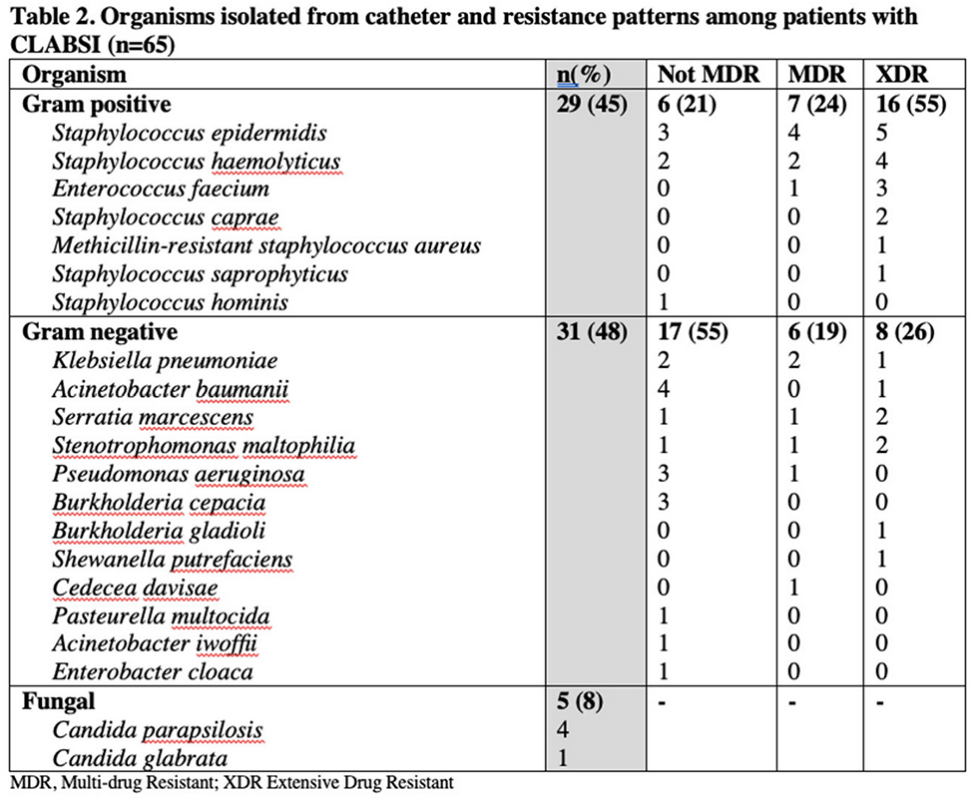

**Methods:**

Retrospective cohort study involving chart review of all admitted patients with central line from January to December, 2023. CLABSI was defined based on the National Health and Safety Network.

**Figure:**
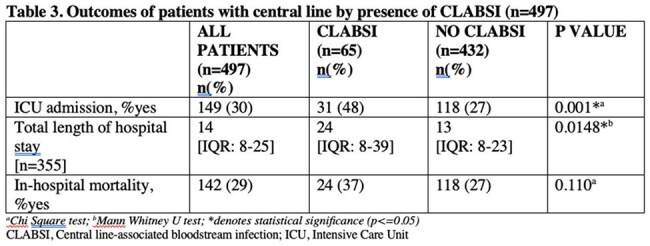

**Figure:**
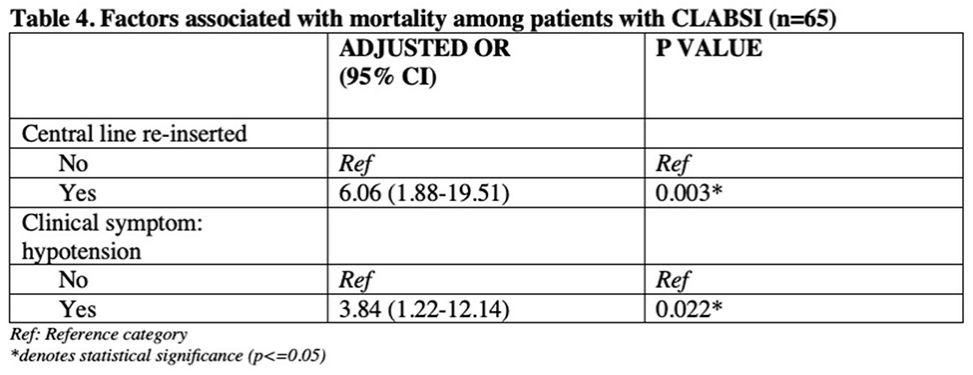

**Results:**

CLABSI was detected in 65 (incidence rate of 13.1%, 95% CI: 10.2-16,4%) out of 497 patients. Fever was the most common presenting symptom (56.92%). A higher proportion of patients with CLABSI had femoral and subclavian central lines than those with no CLABSI (p=0.009). Blood transfusion and central line reinsertion also contributed to CLABSI. Staphylococcus epidermidis was the most common isolate followed by Staphylococcus haemolyticus. Majority of gram positive organisms were found to be extensive drug resistant (55%). CLABSI patients had increased ICU admission (p=0.001) and longer hospital stay (p=0.0148) compared to those who did not develop CLABSI. Mortality was also higher in CLABSI patients; however, was not statistically significant (37% vs 27%, p=0.110). Factors associated with in-hospital mortality included central line reinsertion (aOR=6.06, p=0.003) and hypotension (aOR=3.84, p=0.022).

**Conclusion:**

In this study, we found that incidence of patients developing CLABSI was lower compared to published studies with gram positive organisms as the most common isolates. While it is completely preventable, it is associated with increased mortality and longer hospital stay hence prompt removal of central line is suggested when not in need anymore.

**Disclosures:**

All Authors: No reported disclosures

